# iCD8α cells: living at the edge of the intestinal immune system

**DOI:** 10.18632/oncotarget.4782

**Published:** 2015-07-03

**Authors:** Danyvid Olivares-Villagómez, Luc Van Kaer

**Affiliations:** Department of Pathology, Microbiology and Immunology, Vanderbilt University School of Medicine, Nashville, TN, USA

**Keywords:** Immunology and Microbiology Section, Immune response, Immunity

In order to survive, organisms must distinguish the dangers and growth opportunities that their surrounding environments provide. Food products and microbes are part of these environments and constitute a constant challenge. In humans and most other vertebrates food and microbes have easy access to the inside of the organism by simply entering the gastrointestinal tract. It is here that persistent encounters between the host's immune defenses and outside intruders take place. Although the nature of these encounters varies, there is one common aspect to all of them: the outside environment is separated from the inside tissues by a thin, single layer of epithelial cells known as the intestinal epithelium. Although this monolayer of intestinal epithelial cells (IEC) has a potent barrier function, it does not provide sufficient protection to the organism by itself. This barrier is supported by an extensive network of cells, tissues and organs that interconnect, in one way or another, with the intestinal epithelium. This support system is known as the intestinal mucosal immune system.

In between the IEC reside a large number of lymphoid cells known as intraepithelial lymphocytes (IEL) [1]. IEL are at the edge of the mucosal immune system and are therefore considered sentinels and early responders to microbial invaders. IEL constitute a diverse group of immune cells with distinct functions. Many IEL are T lymphocytes of the adaptive immune system that express a T cell receptor (TCR) comprised of either αβ or γδ chains, whereas others lack TCR expression and belong to the innate arm of the immune system. Although extensive research has been focused on defining the functions and developmental origins of TCR_+_ IEL, little is known about innate-type, TCR_−_ IEL.

Recent studies in immunology have focused on subsets of innate lymphoid cells (ILC) [2], and several of these cell types have been identified within the IEL compartment. For example, this compartment contains an ILC population that expresses the natural killer (NK) cell markers NKp46 and NK1.1, and produces the anti-viral and pro-inflammatory cytokine interferon (IFN)-γ [3]. Additionally, our research group recently identified another TCR_-_ IEL population characterized by expression of CD8α homodimers [4]. This novel subset of lymphoid cells, which we have called iCD8α cells, possesses many attributes associated with innate immune effector functions, such as the capacity to produce pro-inflammatory cytokines and chemokines, exhibit cytotoxicity, engulf and kill bacterial pathogens, and present antigens to MHC class II-restricted T cells. Collectively, these recent findings have revealed that the IEL compartment contains multiple populations of both TCR_+_ and TCR_-_ lymphoid cells with diverse functions.

What is the functional relevance of iCD8α cells? Based on their anatomical location, iCD8α cells would be expected to interact with microorganisms present in the lumen of the intestine, especially those microbes that may directly damage the epithelium. Indeed, we reported that iCD8α cells were able to control colonization of the mouse colon by *Citrobacter rodentium*, a bacterial organism that serves as a model for the human pathogen *Escherichia coli* [4]. We demonstrated that iCD8α cells are capable of engulfing and killing *C. rodentium* bacteria *ex vivo*, raising the possibility that iCD8α cells control pathogenic microbes through this mechanism. Additionally, iCD8α cells may be involved in the homeostasis of commensal microorganisms. Owing to their antigen-presenting properties, combined with their capacity to engulf bacteria, it is tempting to speculate that iCD8α cells can present antigens to TCR_+_ IEL and help orchestrate immune responses in the intestinal epithelium.

Because of their intimate relationship, iCD8α cells likely engage in reciprocal interactions with IEC. For example, IEC produce the cytokine IL-15, which we have shown is critically important for the development and survival of iCD8α cells [4]. Additionally, IEC express the thymus leukemia (TL) antigen (encoded by the *H2-T3* gene), a non-classical MHC class I molecule that functions as a high affinity ligand of the CD8α homodimer [5, 6]. TL expression was previously shown to play an inhibitory role in CD8αα_+_TCR_+_ IEL activation [5, 7, 8], and this molecule may similarly influence the effector functions of iCD8α cells. Conversely, our findings showed that iCD8α cells express high amounts of granzymes A and B, suggesting that these cells exhibit cytotoxic properties, which may be directed against IEC. Possible conditions in which this may occur include infection or transformation of IEC.

In unpublished studies we have further found that iCD8α cells contribute to the development of innate colitis induced by antibodies against the co-stimulatory molecule CD40. We also identified human equivalents to these cells, which were partially depleted in newborns with necrotizing enterocolitis, a condition mostly seen in premature infants [4]. These findings therefore imply an important contribution of iCD8α cells to anti-microbial immunity and colitis.

Living at the edge of the intestinal immune system, iCD8α cells are continuously exposed to foreign substances and microbes. Studies thus far have shown that these cells contribute to providing a first line of defense against microbial pathogens. Future studies will no doubt unveil additional functions of these cells in promoting immune and tissue homeostasis in the delicate microenvironment of the gut mucosa.

## References

[1] Cheroutre H (2011). Nat Rev Immunol.

[2] Eberl G (2015). Science.

[3] Fuchs A (2013). Immunity.

[4] Van Kaer L (2014). Immunity.

[5] Leishman AJ (2001). Science.

[6] Olivares-Villagómez D, Van Kaer L (2010). Immunol Lett.

[7] Olivares-Villagómez D (2008). Proc Natl Acad Sci USA.

[8] Olivares-Villagómez D (2011). J Immunol.

